# Revisiting COVID-19 Communication in Western Africa: A Health Literacy-based Approach to Health Communication

**DOI:** 10.4269/ajtmh.21-0013

**Published:** 2021-07-19

**Authors:** Bernard Seytre, Cristano Barros, Philip Bona, Babacar Fall, Blahima Konaté, Amabelia Rodrigues, Octávio Varela, Marcel Blé Yoro

**Affiliations:** 1 bnscommunication, Paris, France;; 2 Universidade de Cabo Verde, Cabo Verde;; 3 Association for Sustainable Development, Freetown, Sierra Leone;; 4 West African Health Organization, Abuja, Nigeria;; 5 Institut des Sciences des Sociétés, Centre Muraz, Bobo-Dioulasso, Burkina Faso;; 6 Bandim Health Project, Bissau, Guinea Bissau;; 7 Universidade de Cabo Verde, Cabo Verde;; 8 Université Houphouët-Boigny, Abidjan, Ivory Coast

## Abstract

Adherence to protective measures is a major component of COVID-19 epidemic control. COVID-19 health literacy is a major driver of this adherence, and the evaluation of health literacy levels is the basis for designing an effective communication strategy. We conducted a quantitative socio-anthropological study of the knowledge of the severe acute respiratory syndrome coronavirus 2 (SARS-CoV-2) infection and perception of the prevention messages in Burkina Faso, Cabo Verde, Guinea-Bissau, Ivory Coast, and Sierra Leone. There are widespread erroneous ideas regarding the transmission of and the protection against COVID-19. The majority of people are unaware that asymptomatic individuals can transmit the virus. Knowledge of the risk factors for severe disease is not sufficient, and the majority of individuals fear contracting COVID-19 by visiting a health center. Our study also shows the achievements of communication campaigns on several aspects: almost everybody has heard of the virus and heard or read the messages on the protective measures and a large majority of people think that these measures are effective against COVID-19. Based on these results, we propose a communication strategy that will emphasize that asymptomatic individuals can transmit the virus, emphasize the risk factors, reassure individuals regarding the safety of frequenting health centers, and design specific messages targeting young populations.

## INTRODUCTION

Adherence of the population to the prevention measures is a major component of COVID-19 pandemic control, and governments have devoted substantial efforts to disseminating messages that encourage people to respect the protective measures (wearing masks, washing hands, practicing physical distancing, and avoiding hugging and shaking hands). At the same time, an infodemic involving the rapid spread of rumors and false information that could interfere with adherence to the protective measures has developed.^1^

The West Africa Health Organization (WAHO), which is the health agency of the Economic Community of West African States (ECOWAS), requested an analysis of the efficacy of COVID-19 communications and recommendations for an adapted communication strategy. To evaluate the impact of COVID-19 communications and create an objective basis for an eventual new communication strategy, we conducted a quantitative socio-anthropological study of COVID-19 health literacy including the knowledge of the coronavirus and the perception of and adherence to messages regarding protective measures. In agreement with the WAHO, we chose five ECOWAS countries, Cabo Verde, Burkina Faso, Guinea-Bissau, Ivory Coast, and Sierra Leone, because of their official language diversity (two French-speaking countries, two Portuguese-speaking countries, and one English-speaking country), variations in size, economic development, and geographic locations. Additionally, we chose Sierra Leone because of its previous experience with an Ebola virus disease epidemic.

## METHODS

From October 16 to November 20, 2020, we conducted a quantitative socio-anthropological study in Cabo Verde, Burkina Faso, Guinea-Bissau, Ivory Coast, and Sierra Leone. We conducted in-person interviews with 400 people in urban areas of each country, with a breakdown by sex and age groups according to the local demography (Supplemental Table 1). Interviewees were selected randomly during door-to-door visits.

The first household was chosen at random, and then one household out of six was surveyed. Only one person was interviewed in each household; that person was chosen at random if several people met the criteria. The exclusion criteria were mental disability, senility, and any disease that made a person unable to answer the questions. All responses were anonymous. Because all respondents were adults and there were no questions pertaining to their health, we did not submit our protocol to an ethics board.

The questionnaire contained no open-ended questions. Although some questions asked only for “yes,” “no,” or “no answer” responses, most questions provided the respondents with a list of choices with possible responses of “yes,” “no,” or “no answer.” To limit possible confusion between asymptomatic and symptomatic infections and between the coronavirus and COVID-19, we used the expression “COVID-19 disease” when asking about the disease and the term “the coronavirus” when asking about the virus.

The responses were collected on paper and later entered in an online platform by the investigators, or they were directly entered online via tablets using Microsoft Forms. Then, the results were generated and analyzed in an Excel spreadsheet.

A total of 196 people declined to answer, mainly in Sierra Leone (152 people). In Sierra Leone, the nonrespondents who provided an explanation why they declined asserted that they felt they were being used to make money without any benefit to them personally, or that the government was only looking for data to seek funding from the international community.

## RESULTS

The “no answer” responses are not reported here. Therefore, the totals provided may not equal 100%.

### Reality, causal agents, and symptoms of COVID-19.

A limited number of people interviewed thought that COVID-19 does not exist, with substantial differences between countries: 2.7% in Cabo Verde; 9.2% in Guinea-Bissau; 10.7% in Burkina Faso; 18.0% in Ivory Coast; and 20.2% in Sierra Leone. This belief was higher among those with the lowest level of education: 19.7% of interviewees with no education and 15.4%, 9.6%, and 10.6%, respectively, of people with primary, secondary, and superior education.

An average of 97.60% of interviewees had “heard of the coronavirus,” and 78.7% answered “yes” to the question, “Do you know what a virus is?” (Supplemental Table 2).

Although 48.0% to 94.0% of interviewees knew that the virus is found in people who have COVID-19, less than half knew that it can also be found in asymptomatic people (Supplemental Table 3). Similarly, the majority thought that only symptomatic people can transmit the disease. When asked “Who could transmit COVID-19 to you?”, 85.0% (Sierra Leone) to 95.2% (Cabo Verde) answered “yes” to the choice “A person who is ill”; however, only 3.2% to 39.7% answered “yes” to the choice “A person who is not ill” (Supplemental Table 4). Fewer people thought that a human who is not ill can transmit the virus than an animal, an egg, or raw meat (Table 1).

**Table 1:** Positive responses to “Who could transmit COVID-19 to you?”

	Person who is not ill	Eggs	Farm animals	Dog or cat	Mosquitoes	Fish	Wild animals	Raw meat	Ill person
Burkina Faso	39.7%	32.2%	40.5%	42.5%	24.5%	30.5%	67.5%	60.5%	94.5%
Cabo Verde	37.0%	31.2%	20.5%	19.5%	16.5%	46.5%	27.2%	49.5%	95.2%
Ivory Coast	15.5%	8.7%	15.7%	24.0%	22.0%	5.7%	34.7%	21.5%	88.7%
Guinea-Bissau	5.0%	43.2%	39.7%	40.2%	57.2%	60.0%	43.0%	56.0%	91.2%
Sierra Leone	3.2%	1.0%	11.7%	3.2%	22.0%	1.5%	21.0%	7.2%	85.0%
Total	20.1%	23.3%	25.6%	25.9%	28.4%	28.8%	38.7%	38.9%	90.9%

Although cough, headache, fever, and difficulty breathing are well-known symptoms of COVID-19 (Table 2), apart from the respondents in Cabo Verde, the two symptoms most specific to the disease, loss of smell and loss of taste, were known by only 43.5% to 60.5% of the interviewees (Table 3).

**Table 2:** Responses to “Which of the following symptoms may be due to COVID-19?” (average of the five countries)

	No	Yes
Cough	8.1%	86.6%
Headache	16.6%	76.5%
Fever	9.5%	84.7%
Difficulty breathing	7.9%	85.9%
Loss of smell	25.1%	61.8%
Loss of taste	27.0%	60.5%

**Table 3:** Responses to “Which of the following symptoms may be due to COVID-19?”

	Loss of smell		Loss of taste	
	No	Yes	No	Yes
Burkina Faso	35.5%	57.0%	35.5%	57.2%
Cabo Verde	5.5%	89.7%	8.2%	88.5%
Ivory Coast	25.7%	56.5%	30.0%	53.0%
Guinea-Bissau	15.2%	60.5%	16.5%	60.5%
Sierra Leone	43.5%	45.2%	45.0%	43.5%
Total	25.1%	61.8%	27.0%	60.5%

### Prevention and treatment of COVID-19.

Regarding a series of communication messages, including some erroneous ones, we asked interviewees, “Have you heard or seen the following COVID-19 message?”; if they answered “yes,” then we asked a second question, “Do you think this is useful for avoiding COVID-19?”

Almost all interviewees (from 93.2% to 99.7% depending on country and message) had heard or seen messages promoting 1-m distancing, avoidance of shaking hands, washing hands, wearing masks, and avoiding crowds.

We calculated the results to the questions about the usefulness of each measure based on the totals of the interviewees. For example, in Ivory Coast, 96.2% had heard/seen the message about 1-m distancing; of those, 74.5% thought it is useful. Therefore, we calculated that 71.7% (96.2% × 74.5%) found the measure useful. The results were high. Burkina Faso, Guinea-Bissau, and Cabo Verde had an average score of > 90% for the five measures, and Sierra Leone and Ivory Coast had average scores of 82.9% and 75.9%, respectively (Table 4).

**Table 4:** Percentages of interviewees who thought the measure “is useful for avoiding COVID-19”

	Washing hands	Distancing 1 m	Wearing masks	Avoiding crowds	No shaking hands	Average
Ivory Coast	81.3%	71.7%	75.7%	73.8%	76.8%	75.9%
Sierra Leone	81.1%	82.5%	83.6%	84.3%	83.0%	82.9%
Burkina Faso	93.78%	90.3%	87.1%	91.0%	91.5%	90.7%
Guinea-Bissau	95.8%	92.1%	95.5%	90.5%	91.6%	93.1%
Cabo Verde	99.0%	98.0%	98.3%	98.0%	97.3%	98.1%

We asked the question, “Do the following circumstances or actions reduce the risk of getting COVID-19?” and provided 14 possible answers. The overall average number of respondents who believed that some protection is conferred by eating garlic or drinking lemon juice, hot drinks, tea, bleach, disinfectant, and neem leaf infusions varied from 14.3% in Burkina Faso to 51.0% in Guinea-Bissau (Table 5). “Drinking a disinfectant” was considered to offer protection by 12.0% in Sierra Leone and by 48.2% in Guinea-Bissau. Similarly, “drinking bleach” was considered to offer protection by 10.0% in Sierra Leone, 14.2% in Cabo Verde, and 64.7% in Guinea-Bissau. Regarding drinking tea, the positive answers ranged from 13.7% in Burkina Faso to 59.2% in Guinea-Bissau.

**Table 5:** Positive responses to “Do the following reduce the risk of getting COVID-19?”

	Drinking lemon juice	Eating garlic	Hot drinks	Drinking tea	Drinking bleach	Drinking disinfectant	Drinking an infusion of neem leaves	Average
Burkina Faso	18.7%	3.2%	28.7%	13.7%	3.5%	3.2%	28.7%	14.3%
Cabo Verde	53.5%	1.2%	23.5%	56.7%	14.2%	1.2%	23.5%	24.9%
Ivory Coast	14.2%	3.5%	30.7%	19.0%	1.5%	3.5%	30.7%	14.7%
Guinea-Bissau	55.5%	48.2%	40.5%	59.2%	64.7%	48.2%	40.5%	51.0%
Sierra Leone	27.2%	12.0%	12.5%	15.5%	10.0%	12.0%	12.5%	14.5%

### Disease risks.

An average of 63.8% of respondents knew that people older than 60 years are at higher risk for COVID-19; however, there were differences between countries. Fewer than half of the interviewees were aware of this risk in Cabo Verde and Sierra Leone (Supplemental Table 5).

Similarly, we asked whether people with diabetes, hypertension, heart disease, lung disease, and excessive weight are at higher risk, the same risk, or lower risk for “being seriously ill with COVID-19.” The risks engendered by hypertension and heart disease, as well as lung disease, were known by more than two-thirds of interviewees, except in Sierra Leone. The risk of excessive weight/obesity was known by fewer than half of the interviewees. Knowledge of the risk factors was strikingly lower in Sierra Leone than in the other countries (Supplemental Table 6).

### Distrust of health centers.

To evaluate whether fear of contracting COVID-19 could prevent people from presenting to health centers, we asked the question “Can you catch COVID-19 by visiting a health center for a reason other than COVID-19?” On average, a majority answered “Yes,” with a range from 41.0% in Sierra Leone to 92.5% in Cabo Verde (Table 6).

**Table 6:** Perceptions of the possibility of acquiring COVID-19 at a health center

	No	No response	Yes
Burkina Faso	15.2%	2.2%	82.5%
Cabo Verde	5.5%	2.0%	92.5%
Ivory Coast	26.7%	11.5%	61.7%
Guinea-Bissau	33.2%	8.7%	58.0%
Sierra Leone	45.5%	13.5%	41.0%
Total	25.2%	7.6%	67.1%

### COVID-19 risk perception by the youth.

The perceived risk of illness attributable to COVID-19 was significantly different among age groups. Interviewees in the 18 to 24 years age group were more aware than those in the 60 years and older age group that elderly people are at higher risk for COVID-19 than young people (Tables 7 and 8): 56.8% of the respondents in the 18 to 24 years age group knew they are at lower risk for COVID-19, and 70.1% knew that elderly people are at higher risk.

**Table 7:** Perceived COVID-19 risk for people 18 to 24 years of age

Age of interviewee, years	Lower risk	Same risk	Higher risk
18–24	56.8%	25.2%	6.3%
25–59	47.0%	30.8%	12.0%
60 or older	37.1%	37.1%	11.7%
Total	49.3%	29.5%	10.0%

**Table 8:** Perceived COVID-19 risk for people 60 years or older

Age of interviewees, years	Less risk	Same risk	More risk
18–24	4.8%	18.0%	70.1%
25–59	7.1%	22.6%	63.0%
60 or older	10.1%	30.5%	47.2%
Total	6.6%	21.8%	63.8%

## DISCUSSION

To our knowledge, this study is the only quantitative field survey of the knowledge of SARS-CoV2 infection, the perception of prevention messages, and the effectiveness of the protective measures in sub-Saharan Africa.

Many false ideas of the possible ways of contracting COVID-19 are circulating. Approximately one-third of the population thinks that mosquitoes or wild animals can transmit COVID-19. The origin of the human coronavirus causing COVID-19 is a bat species inhabiting an Asian area; however, the fact that other diseases present in Africa are transmitted by mosquitoes or wild animals may explain these erroneous ideas.^2^

It is more surprising that a significant number of people think that raw meat, fish, cats, dogs, and eggs can transmit the disease. COVID-19 appears to be the first disease with an origin attributed to these animals or animal products. During the first 5 or 6 months of the pandemic in Africa, erroneous messages regarding the supposed risk of transmission from these animals and animal products were disseminated by national and international health authorities.^3^ Beliefs in these erroneous risks are strikingly lower in countries where these messages were not disseminated than in countries where they were disseminated: 1.0% and 1.5% for eggs and fish, respectively, in Sierra Leone; 8.7% and 5.7% for eggs and fish, respectively, in Ivory Coast; and 30.5% to 60.0% for eggs and fish, respectively, in the four other countries (some messages regarding eggs were disseminated in Ivory Coast).

A substantial number of people indicated that they believe that various plants or substances protect against COVID-19. Although garlic, lemon juice, neem leaf infusions, and hot drinks are often considered protective against various diseases in African cultures, it seems that drinking bleach or disinfectant has never been considered protective or curative previously.^4^^,^^5^ Some people might have become confused about using and drinking disinfectant; however, we can hypothesize that these incorrect ideas originated from the widely broadcast declarations by President Trump in the United States, who believed that swallowing these products could be beneficial.^6^ However, we have no explanation for the differences between the percentages of people who believed these measures were protective in the five countries of our study. This could be because of differences in the circulation intensity of these declarations, which we have not evaluated.

Believing in unfounded risks for contracting COVID-19 could cause populations to relativize the only proven route of infection in Africa, interhuman transmission. Similarly, believing in erroneous protection measures could generate a false feeling of safety that could hamper adherence to protective measures.

The fact that some of the erroneous ideas originated from erroneous communication messages highlights the effectiveness of the penetration of communication messages and the responsibility of health communicators and politicians to verify the accuracy of their communications. Certain studies have indicated that a significant portion of the population does not respect the protective measures (washing hands, wearing masks, physical distancing).^7^^–^^11^ Observations by our team of investigators that were documented by photographs showed that wearing masks and physical distancing are not sufficiently respected by the populations in Burkina Faso, Guinea-Bissau, Ivory Coast, and Sierra Leone. However, our results showed that almost everyone has heard or read the messages promoting the protective measures, and that the majority of people think that these measures effectively prevent COVID-19. Therefore, nonadherence to these measures is not attributable to a lack of knowledge or a lack of understanding, and we cannot expect that repetition of these messages alone would change the behaviors.

The significant lack of understanding of the coronavirus infection history revealed by our study is likely contributes to the insufficient adherence to the protective measures. First, although people have heard of coronavirus and the majority reported knowing what a virus is, the majority think only sick persons can transmit the virus. Animals and animal products are considered a much greater risk for COVID-19 transmission than asymptomatic individuals. How can we make people understand that everybody must respect the protective measures if they think that only sick individuals can transmit the virus? How can we make people adhere to these measures when they think that an animal or an egg is more likely to transmit the virus than people next to them on a bus? According to the WHO, 80% of SARS-CoV2 infections in sub-Saharan Africa are asymptomatic.^12^ A major communication effort must be organized to explain that most of the people infected by the coronavirus transmit it to others without ever showing any symptoms. Second, the risk factors for serious forms of COVID-19 (older age, diabetes, cardiovascular diseases, and excessive weight) are not sufficiently known, and there are differences between countries and risk factors. A communication effort regarding the risk of being overweight in all countries and on all risk factors in Ivory Coast and especially Sierra Leone must be developed. Knowledge of these risks would provide motivation for adopting the protection measures when in the presence of someone at risk. Third, young populations are aware that they are at lower risk for COVID-19 than older people; therefore, specific messages should be designed to convince them to adopt the protective measures. In addition to explaining that one can be asymptomatic and still transmit the coronavirus, as discussed, these messages should call on their responsibility to protect friends and family members who are at risk because of chronic disease or age. Fourth, our results show deep concern among people regarding contracting COVID-19 at health centers. This is likely to contribute to the observed decrease in the prevention, diagnosis, and treatment of diseases other than COVID-19.^13^^–^^15^ A major communication effort must be developed to reassure people and restore their frequenting of health centers to normal levels.

## CONCLUSION

This quantitative study conducted in five Western African countries showed that 9 months into the COVID-19 pandemic, communication efforts have succeeded in transmitting certain major elements of knowledge regarding the disease; however, other critical elements that could allow the population to fully understand the necessity of the protective measures are missing. Therefore, the communication efforts must be redirected to no longer focus on the protective measures, which people know about but do not sufficiently practice. Instead, efforts must be focused on the missing information and misunderstandings that our study has revealed. A good COVID-19 health literacy level is necessary for full adherence to these measures.^16^ Developing a new strategy to increase this level is one of the challenges of communication. Therefore, we propose the following four-point communication strategy:

Explain that asymptomatic people can transmit the virus.

Develop knowledge of the risk factors for serious illness.

Target young populations with appropriate messages.

Reassure populations that it is safe to frequent health centers.

The results of this study and the consequent communication strategy are likely to be applicable to other non-ECOWAS sub-Saharan countries.

## Supplemental tables


Supplemental materials
