# Supplementary material for: Revisiting COVID-19 Communication in Western Africa: A Health Literacy-based Approach to Health Communication
Source: Am J Trop Med Hyg. 2021 Jul 19;105(3):708–12. doi: 10.4269/ajtmh.21-0013 (PMC8592366; doi:10.4269/ajtmh.21-0013)
Supplement: Supplementary file 1 [file tpmd210013.SD1.docx]

**Supplemental Table 1: Interviewees by sex, age groups, educational level and occupation.**

|  | Burkina Faso | Cabo Verde | Ivory Coast | Guinea-Bissau | Sierra Leone | Total |
| --- | --- | --- | --- | --- | --- | --- |
| **Females** | **206** | **199** | **192** | **215** | **195** | **1007** |
| 18-24 | 140 | 37 | 85 | 53 | 32 | 347 |
| 25-59 | 55 | 127 | 101 | 140 | 139 | 562 |
| 60 + | 11 | 35 | 6 | 22 | 24 | 98 |
| **Males** | **194** | **201** | **208** | **185** | **205** | **993** |
| 18-24 | 131 | 41 | 83 | 39 | 42 | 336 |
| 25-59 | 50 | 134 | 109 | 130 | 135 | 558 |
| 60 + | 13 | 26 | 16 | 16 | 28 | 99 |
| **Education** |  |  |  |  |  |  |
| None | 69 | 28 | 72 | 54 | 67 | 290 |
| Primary | 85 | 73 | 49 | 57 | 48 | 312 |
| Secondary | 204 | 230 | 119 | 198 | 168 | 919 |
| Superior | 42 | 69 | 160 | 91 | 117 | 479 |
| **Occupation** |  |  |  |  |  |  |
| Formal private sector | 22 | 72 | 68 | 46 | 39 | 247 |
| Formal public sector | 11 | 83 | 31 | 47 | 38 | 210 |
| Informal sector | 155 | 72 | 147 | 144 | 111 | 629 |
| No activity | 38 | 84 | 50 | 72 | 85 | 329 |
| Retired | 3 | 34 | 9 | 7 | 22 | 75 |
| Student | 171 | 55 | 95 | 84 | 105 | 510 |
| **Total** | **400** | **400** | **400** | **400** | **400** | **2000** |

**Supplemental Table 2: “Do you know what a virus is?”**

|  | **No** | **Yes** |
| --- | --- | --- |
| Burkina Faso | 20.7% | 79.2% |
| Cabo Verde | 13.2% | 86.7% |
| Ivory Coast | 18.2% | 81.7% |
| Guinea-Bissau | 34.5% | 65.5% |
| Sierra Leone | 19.5% | 80.5% |
| **Total** | **21.2%** | **78.7%** |

**Supplemental Table 3: Responses to the question “In whom can we find the virus?”**

|  | People ill with COVID-19 | | | Everyone | | |
| --- | --- | --- | --- | --- | --- | --- |
|  | N/P | No | Yes | N/P | No | Yes |
| Burkina Faso | 4.5% | 47.5% | 48.0% | 4.5% | 45.5% | 50.0% |
| Cabo Verde | 1.0% | 5.0% | 94.0% | 4.7% | 60.2% | 35.0% |
| Ivory Coast | 8.2% | 35.0% | 56.7% | 10.7% | 42.0% | 47.2% |
| Guinea-Bissau | 11.2% | 4.0% | 84.7% | 12.0% | 77.7% | 10.2% |
| Sierra Leone | 11.0% | 6.2% | 82.7% | 18.0% | 67.7% | 14.2% |
| **Total** | **7.2%** | **19.5%** | **73.2%** | **10.0%** | **58.6%** | **31.3%** |

**Supplemental Table 4: “Who could transmit COVID-19 to you?” Responses to “A person who is not ill.”**

|  | No | Yes |
| --- | --- | --- |
| Burkina Faso | 57.5% | 39.7% |
| Cabo Verde | 60.0% | 37.0% |
| Ivory Coast | 77.0% | 15.5% |
| Guinea-Bissau | 90.0% | 5.0% |
| Sierra Leone | 84.7% | 3.2% |
| **Total** | **73.8%** | **20.1%** |

**Supplemental Table 5: What is the risk of becoming ill with COVID-19 for people aged 60 years or more?**

|  | Less risk | Same risk | More risk |
| --- | --- | --- | --- |
| Burkina Faso | 2.2% | 22.5% | 70.7% |
| Cabo Verde | 5.5% | 46.5% | 46.2% |
| Ivory Coast | 2.2% | 14.2% | 76.5% |
| Guinea-Bissau | 3.5% | 7.0% | 78.5% |
| Sierra Leone | 19.7% | 18.7% | 47.2% |
| **Total** | **6.6%** | **21.8%** | **63.8%** |

**Supplemental Table 6: Positives responses to “more risk of being serious ill with COVID-19.”**

|  | Diabetes | AHT and CVD | Lung disease | Overweight | Average |
| --- | --- | --- | --- | --- | --- |
| Burkina Faso | 86.0% | 85.0% | 86.7% | 53.5% | 77.8% |
| Cabo Verde | 89.2% | 90.2% | 90.5% | 57.7% | 81.9% |
| Ivory Coast | 57.7% | 67.0% | 72.5% | 42.2% | 59.9% |
| Guinea-Bissau | 78.7% | 77.7% | 81.0% | 66.2% | 75.9% |
| Sierra Leone | 24.0% | 32.2% | 32.0% | 16.5% | 26.2% |
| **Total** | **67.1%** | **70.4%** | **72.5%** | **47.2%** | **64.3%** |
|  |  |  |  |  |  |
